# Long Noncoding RNAs in Myocardial Ischemia-Reperfusion Injury

**DOI:** 10.1155/2021/8889123

**Published:** 2021-04-05

**Authors:** Zhuo Zhao, Wei Sun, Ziyuan Guo, Bin Liu, Hongyu Yu, Jichang Zhang

**Affiliations:** ^1^Department of Cardiology, The Second Hospital of Jilin University, 218 Ziqiang Road, Changchun, China 130041; ^2^Department of Nephrology, The Second Hospital of Jilin University, 218 Ziqiang Road, Changchun, China 130041

## Abstract

Following an acute myocardial infarction, reperfusion therapy is currently the most effective way to save the ischemic myocardium; however, restoring blood flow may lead to a myocardial ischemia-reperfusion injury (MIRI). Recent studies have confirmed that long-chain noncoding RNAs (LncRNAs) play important roles in the pathophysiology of MIRIs. These LncRNA-mediated roles include cardiomyocyte apoptosis, autophagy, necrosis, oxidative stress, inflammation, mitochondrial dysfunction, and calcium overload, which are regulated through the expression of target genes. Thus, LncRNAs may be used as clinical diagnostic markers and therapeutic targets to treat or prevent MIRI. This review evaluates the research on LncRNAs involved in MIRIs and provides new ideas for preventing and treating this type of injury.

## 1. Introduction

An acute myocardial infarction (AMI) is a type of cardiovascular event that seriously endangers human health [1, 2]. Timely reperfusion treatment following the onset of an AMI can restore blood flow, salvage viable myocardium, reduce the myocardial infarction size, and preserve left ventricular (LV) systolic function [3–5]. However, the process of myocardial reperfusion can further aggravate ischemic myocardium damage, leading to a myocardial ischemia-reperfusion injury (MIRI). Clinical presentations of a MIRI are reperfusion-induced arrhythmias, myocardial stunning, microvascular obstruction, and lethal myocardial reperfusion injury [6–8]. Protecting the heart from the detrimental effects of a MIRI is a therapeutic challenge for physicians. Therefore, elucidating the key mechanisms that prevent a MIRI would greatly aid in the development of an effective therapy for AMIs. Recent studies have shown that the mechanisms of a MIRI include cardiomyocyte apoptosis, autophagy, necrosis, oxidative stress, inflammation, mitochondrial dysfunction, and intracellular calcium overload, all of which involve a multitude of signaling pathways and molecular players [6, 7, 9].

Recently, long noncoding RNAs (LncRNAs) have been shown to modulate key interactions with molecular pathways associated with MIRI progression and potentially serve as novel therapeutic targets. LncRNAs are a class of noncoding RNAs longer than 200 nucleotides. LncRNAs can regulate gene expression at multiple levels, including regulation of epigenetic, transcriptional, and posttranscriptional processes. This review discusses recent studies that have examined the functional role of LncRNAs in MIRIs, their therapeutic potential and aspects that should be further evaluated in the future.

## 2. LncRNAs

LncRNAs are a class of noncoding RNAs that are more than 200 nucleotides in length that are typically synthesized via RNA polymerase II. Based on the position of the LncRNA and coding gene, these types of RNAs are divided into five categories: (1) sense LncRNA, (2) antisense LncRNA, (3) intron LncRNA, (4) bidirectional noncoding LncRNA, and (5) intergenic LncRNA. LncRNAs regulate gene expression on both chromatin and DNA at the transcriptional and posttranscriptional level, all of which can affect the occurrence and outcome of various diseases [10]. Some regulatory mechanisms exerted through LncRNAs may involve interfering with the translation of an encoded gene, inhibiting polymerase II activity, facilitating posttranscriptional modification, binding to functional proteins, or serving as a precursor for a small molecule RNA that binds to chromosomes to regulate signaling pathways [11, 12].

In addition to being directly involved in regulating gene expression, LncRNAs can also function as a competing endogenous RNA (ceRNA)—i.e., competes with other RNA transcripts for the same microRNA (miRNA), thereby affecting resulting interactions and subsequent signaling cascades. miRNAs are endogenous noncoding RNAs composed of 21 to 25 nucleotides that mainly facilitate the posttranscriptional regulation of target genes through binding to the 3′ untranslated region (UTR) of the target mRNA [13]. In a study on the interactions between RNA transcripts, Salmena et al. [14] first proposed a ceRNA-centered hypothesis as a novel model to regulate gene expression. In this model, transcripts such as LncRNA, pseudogene transcripts, or mRNAs can serve as ceRNAs through miRNA response elements (MREs), which can competitively bind to miRNAs with the same MRE to regulate gene expression levels, thereby affecting downstream cellular functions. Currently, functional studies on ceRNAs suggest that LncRNAs and miRNAs are mutually regulated via competitive binding to the corresponding MRE, thereby effectively controlling the subsequent posttranscriptional regulation of the miRNAs [15, 16].

## 3. The Role of LncRNAs in MIRIs

**Figure 1:**
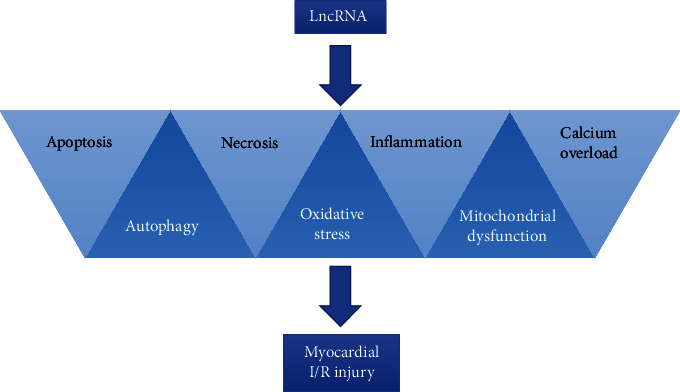
The role of LncRNAs in MIRI.

The relationship between LncRNAs and a MIRI has been reported in several studies. Recently, Wu et al. reported that 2,292 LncRNAs were upregulated and 1,848 LncRNAs were downregulated in patients with a MIRI [17]. Using gene chip analysis in a mouse model of ischemia-reperfusion (I/R), Liu et al. found that 64 LncRNAs were upregulated and 87 LncRNAs were downregulated in the region with the infarction [18]. Similarly, using gene chip analysis in a cell-based study, Huang et al. found that 309 LncRNAs were upregulated and 488 LncRNAs were downregulated following hypoxia/reoxygenation (H/R) in H9c2 cells [19]. Recent studies have provided credible evidence that LncRNAs play a vital role in the initiation and progression of a MIRI through their involvement in apoptosis, autophagy, and necrosis of cardiomyocytes, as well as by affecting oxidative stress, inflammation, mitochondrial dysfunction, and intracellular calcium overload (Figure 1 and Table 1).

### 3.1. LncRNAs and Apoptosis

Cardiomyocyte apoptosis is a key pathophysiological mechanism underlying a MIRI. Apoptosis is a type of programmed cell death induced by physiological or pathological factors. The process is characterized by cell shrinkage, chromatin condensation, and systematic DNA cleavage [20]. Apoptosis is a highly regulated cellular process, which can be divided into two classic pathways: the extrinsic or intrinsic pathway. The extrinsic pathway, also known as the death receptor-associated pathway, is activated via the death-inducing signal complex (DISC). The DISC activates caspase-8, which in turn triggers the downstream activation of executioner caspases, leading to apoptosis. The intrinsic pathway, also known as the mitochondrial pathway, is initiated by increased permeability of the outer mitochondrial membrane (OMM), leading to the translocation of proapoptotic molecules such as cytochrome C (cytC) and apoptosis-inducing factor (AIF) from the mitochondrial intermembrane space to the cytosol. This leads to the formation of the apoptosome complex and subsequent downstream activation of the caspase cascade. Members of the Bcl-2 family are major participants in the intrinsic pathway [20, 21]. In some circumstances, the intrinsic pathway is also initiated via the endoplasmic reticulum stress- (ERS-) induced apoptotic signaling pathway. It has been demonstrated that prolonged and/or excess ERS may lead to substantial apoptosis and be a major contributor to a MIRI. Herein, we expound on the relationships between different LncRNAs and their role during apoptosis in response to a MIRI [22–25].

#### 3.1.1. LncRNA MALAT1

The expression of the LncRNA MALAT1 is significantly increased in the myocardial tissue of the rat I/R model. Knockdown of MALAT1 can markedly improve I/R-induced myocardial contractile dysfunction and inhibit cardiomyocyte apoptosis in the rat I/R injury model by upregulating the expression of *β*-catenin [26]. LncRNA MALAT1 was prominently elevated in oxygen-glucose deprivation/reoxygenation- (OGD/R-) induced H9c2 cells. MALAT1 knockdown inhibited OGD/R-induced cardiomyocyte apoptosis by activating the AKT/GSK-3*β*/*β*-catenin signaling pathway via downregulation of miR-122 [27]. LncRNA MALAT1 expression was upregulated in H/R-treated AC16 cells. Moreover, MALAT1 regulated hypoxia-induced myocardial cell apoptosis by sponging miR-200a-3p to upregulate PDCD4 [28]. The expression of LncRNA MALAT1 and phosphatase and tensin homolog deleted on chromosome 10 (Pten) was upregulated, whereas that of miR-320 was downregulated in myocardial tissues from the AMI mouse model. MALAT1 functions as a ceRNA for miR-320 to regulate Pten in mouse cardiomyocytes. Overexpression of MALAT1 aggravated cardiomyocyte apoptosis *in vitro*, and knockdown of MALAT1 improved cardiac function, resulting from AMI [29].

#### 3.1.2. LncRNA PFL

The expression level of the LncRNA PFL was markedly improved in the myocardial tissues, whereas the level of HSP-20 was markedly decreased in the myocardium of the I/R rat model. HSP-20 can form a stable complex with Bax and to in turn regulate the ratio of Bax/Bcl-2, thereby maintaining mitochondrial integrity and inhibiting both cytC release and caspase-3 activation. Knocking down PFL can inhibit MIRI-mediated apoptosis by upregulating HSP-20 expression [30].

#### 3.1.3. LncRNA HIF1A-AS1

LncRNA HIF1A-AS1 expression was upregulated in the mouse model of I/R. Interestingly, LncRNA HIF1A-AS1 functions as a ceRNA when it binds with miR-204, thereby suppressing its expression and upregulating suppressor of cytokine2 (SOCS2) expression. Members of the SOCS protein family were initially described as cytokine-induced JAK/STAT signaling feedback inhibitors. Silencing HIF1A-AS1 can inhibit I/R-induced apoptosis in cardiomyocytes to alleviate ventricular remodeling following an AMI in mice [31].

#### 3.1.4. LncRNA Nuclear Paraspeckle Assembly Transcript 1 (NEAT1)

LncRNA NEAT1 expression was upregulated in H/R-treated H9c2 cells. LncRNA NEAT1 can bind to miR-520a to suppress its expression. Additionally, NEAT1 knockdown can elevate Bax expression levels, cleave caspase-3, and reduce the expression level of Bcl-2. Knocking down NEAT1 can inhibit cardiomyocyte apoptosis induced via H/R [32]. Another study also reported that NEAT1 plays a crucial role in exacerbating the myocardial injury in MIRI mouse models. Moreover, NEAT1 could prompt cardiomyocyte apoptosis induced via I/R by activating the MAPK signaling pathway [33].

#### 3.1.5. LncRNA Meg3

LncRNA Meg3 expression was markedly upregulated in I/R-treated H9c2 cells and in the I/R rat model. Meg3 can promote cell apoptosis, inhibit cell proliferation, and promote myocardial damage by directly binding to miR-5-7p. *PARP1*, the downstream target gene of miR-7-5p, plays an important role in cardiomyocyte apoptosis. LncRNA Meg3 regulates cardiomyocyte apoptosis in MIRI via the miR-7-5p/PARP1 signaling pathway [34].

#### 3.1.6. LncRNA Myocardial Infarction-Associated Transcript (MIAT)

The expression of MIAT, another LncRNA, significantly increased in H9c2 cells when H/R stimulated as well as in the heart of mice in response to the I/R-induced injury. MIAT can regulate the expression of PUMA via NF-*κ*B activation in H/R-induced H9c2 cells. Subsequently, PUMA plays an important role in inducing cell apoptosis. Silencing LncRNA MIAT could inhibit H/R-induced cardiomyocyte apoptosis and injury by suppressing the NF-*κ*B and PUMA signaling pathways [35].

#### 3.1.7. LncRNA X-Inactive Specific Transcript (XIST)

In H/R-treated AC16 cells, LncRNA XIST expression was upregulated, whereas miR-130a-3p was downregulated. Additionally, XIST can negatively regulate miR-130a-3p expression. *PDE4D* is a direct target gene of miR-130a-3p that promotes cardiomyocyte apoptosis. Depleting LncRNA XIST suppressed apoptosis and promoted cell survival in H/R-treated AC16 cells via the miR-130a-3p/PDE4D signaling pathway [36].

#### 3.1.8. LncRNA AK123484

The expression of LncRNA AK123484 was upregulated in H9c2 cells treated with anoxia/reoxygenation (A/R). LncRNA AK123483 positively correlated with the expression of PARP. Consequently, LncRNA AK123483 knockdown protects cardiomyocytes against A/R-induced apoptosis by inhibiting PARP expression [37].

#### 3.1.9. LncRNA BDNF-AS

LncRNA BDNF-AS expression was upregulated in H/R-treated cardiomyocytes. Knocking down BDNF-AS can inhibit apoptosis and promote cardiomyocyte viability under H/R conditions by upregulating BDNF expression. Therefore, BDNF upregulation exerts its protective effect against MIRIs by activating survival-signaling pathways, including upregulating VEGF and phosphorylating, i.e., activating, AKT [38].

#### 3.1.10. LncRNA H19

H19 is a conserved LncRNA that is transcribed from the imprinted H19-insulin growth factor 2 (IGF2) locus; moreover, it is located in both the nucleus and the cytoplasm. LncRNA H19 was downregulated in the mouse model of I/R and H_2_O_2_-treated cardiomyocytes. Further, H19 acts as a ceRNA to inhibit miR-877-3p expression and suppress Bcl-2 expression. Therefore, expression of miR-877-3p exacerbates H_2_O_2_-induced injuries in cardiomyocytes as well as MIRIs via Bcl-2 through its mediated mitochondrial apoptotic pathway. These effects suggest that miR-877-3p and Bcl-2 serve as downstream mediators of H19 under apoptotic conditions. Further, LncRNA H19 alleviated cardiomyocyte apoptosis by suppressing the miR-877-3p/Bcl-2-mediated mitochondrial apoptotic pathway [39].

#### 3.1.11. LncRNA UCA1

LncRNA UCA1 expression was significantly downregulated, and reactive oxygen species (ROS) levels were significantly enhanced in the rat model of I/R. UCA1 can directly inhibit p27 at the protein level. Overexpression of p27 can activate caspase-3 by promoting its cleavage; moreover, it can trigger H_2_O_2_-induced cardiomyocyte apoptosis. Suppressing UCA1 has a proapoptotic function by regulating p27 protein levels [40].

#### 3.1.12. LncRNA FAF

LncRNA FAF expression is downregulated in cardiomyocytes treated with ischemia-hypoxia as well as the myocardium of AMI rats. Overexpressing LncRNA FAF can inhibit cardiomyocyte apoptosis induced via ischemia and hypoxia. LncRNA FAF positively regulates FGF9 expression. Moreover, FGF9 can specifically activate FGFR2, which is associated with the PI3K/AKT pathway. Knocking down FGF9 can promote cardiomyocyte apoptosis induced by ischemia and hypoxia. We theorize that LncRNA FAF plays a protective role in inhibiting cardiomyocyte apoptosis by upregulation FGF9/FGFR2 via the PI3K/AKT signaling pathway [41].

### 3.2. LncRNAs and Autophagy

Autophagy is an intracellular protective mechanism under physiological conditions that can degrade damaged or unnecessary proteins, as well as organelles, to maintain cellular homeostasis. When a MIRI occurs, autophagy is activated in response to oxidative stress, ERS, and energy deprivation. Recent studies have demonstrated that autophagy can be either protective or detrimental to cardiac tissue during a MIRI event [42, 43]. Autophagy activated during the initial phase of ischemia has protective effects on cardiomyocytes. For instance, during the myocardial ischemia stage, AMPK is activated in response to the decreasing level of ATP, which occurs when a high ratio of AMP/ATP owing to the deprivation of nutrients and oxygen. Therefore, AMPK is an essential molecule to initiate autophagy in cardiac ischemia. AMPK could launch autophagy through the AMPK/mTORC1/ULK1 signaling pathway or by directly activating ULK1. Autophagy promotes cardiomyocyte survival by generating the amino acids and fatty acids required for maintaining cellular energy levels, as well as activating mitophagy, which can prevent damaged mitochondria from releasing their cytotoxic substances. However, uncontrolled excessive autophagy during the reperfusion stage exerts detrimental effects on cardiomyocytes. During the reperfusion phase, increased ROS generation prompts the overexpression of Beclin-1, which is a crucial mediator of autophagy during this phase. Consequently, overexpressing Beclin-1 can enhance autophagic activity during reperfusion. Excessive autophagy degrades essential proteins or organelles, thereby leading to autophagy-mediated cell death. Additionally, apoptosis is caused by detrimental effects of excessive autophagy in MIRI [44, 45].

#### 3.2.1. LncRNA TUG1

LncRNA TUG1 expression was upregulated in I/R-injured heart tissues and H_2_O_2_-challenged cardiomyocytes. TUG1 was identified as a miRNA sponge that targets miR-142-3p, which affects the activity of downstream target molecules, e.g., HMGB1 and Rac1, that contribute to autophagic cell death. Depleting TUG1 could inhibit excessive autophagy-induced cell death and reduce I/R-induced infarction size [46].

#### 3.2.2. LncRNA XIST

LncRNA XIST expression was upregulated in H/R-treated H9c2 cells. Subsequently, XIST increases SOCS2 expression by negatively regulating miR-133a, which results in excessive autophagy in cardiomyocytes. Further, XIST inhibition exerted a myocardial protective effect during a MIRI by suppressing autophagy and regulating the miR-133a/SOCS2 axis [47].

#### 3.2.3. LncRNA-HRIM

The LncRNA-HRIM is located on chromosome 20p12, which includes three exons, and is a total of 1,470 bp. Notably, LncRNA-HRIM is upregulated in H/R-treated H9c2 cells as well as the rat model of I/R. Inhibition of LncRNA-HRIM expression can markedly increase myocardial cell survival by reducing excessive autophagy during H/R [19].

#### 3.2.4. LncRNA AK139328

LncRNA AK139328 expression was evidently upregulated in diabetic mice with a MIRI. LncRNA AK139328 directly modulates miR-204-3p expression. Silencing LncRNA AK139328 can inhibit the expression of autophagy-related proteins (e.g., LC3-I/LC3-II, ATG5, and ATG7) in response to a MIRI. Knocking down AK139328 exerts a protective effect in diabetic mice with a MIRI by inhibiting cardiomyocyte autophagy [48].

#### 3.2.5. LncRNA AK088388

LncRNA AK088388 expression is markedly upregulated in H/R-treated HL-1 cells. AK088388 can regulate LC3-II expression via the miR-30a/Beclin-1 pathway. The upregulated expression of AK088388 activates autophagy and promotes cardiomyocyte injury in response to H/R. Thus, AK088388 knockdown could promote cell viability and prevent extensive damage in cardiomyocytes [49].

#### 3.2.6. LncRNA FOXD3-AS

LncRNA FOXD3-AS expression is upregulated in OGD/R-treated H9c2 cells. Overexpression of FOXD3-AS1 can increase the expression of autophagy-associated proteins (LC3II, Beclin-1, and ATG5) and reduce the expression of p62. Moreover, FOXD3-AS1 overexpression also augments the expression of phosphorylated (p)-NF-*κ*B, p65, COX-2, and INOS. FOXD3-AS1 aggravates the I/R-induced injury in cardiomyocytes through excessive autophagy that is regulated by activating the NF-*κ*B/iNOS/COX2 signaling pathway [50].

#### 3.2.7. LncRNA RMRP

The expression of LncRNA RMRP is increased in hypoxia-treated H9c2 cells as well as in the rat model of I/R. RMRP negatively regulates the expression of miR-206; however, RMRP overexpression can activate the autophagic response and aggravate subsequent cell damage by downregulating miR-206. Moreover, overexpression of miR-206 has a protective effect on hypoxia-induced injuries by targeting the regulation of ATG3 expression. Overexpression of RMRP activates the PI3K/AKT/mTOR signaling pathway, which is reversed following miR-206 overexpression. The RMPR/miR-206/ATG3 axis can mediate cell autophagy by regulating the activation of PI3K/AKT/mTOR signaling in response to a MIRI [51].

#### 3.2.8. LncRNA AK079427

The expression of the LncRNA AK079427, also known as autophagy-promoting factor (APF), was significantly upregulated in A/R-treated cardiomyocytes and the mouse model of I/R. APF specifically sponges miR-188-3p to regulate its activity. Overexpression of miR-188-3p can inhibit both autophagy and myocardial infarction by downregulating its downstream target, *ATG7*. *ATG7* is a key autophagy promoting gene that encodes an E1-like enzyme, a member of the autophagy system. APF knockdown can attenuate cardiomyocyte autophagy and reduce myocardial infarction sizes in response to a I/R injury by targeting the miR-188-3p/ATG7 axis. Thus, the APF/miR-188-3p/ATG7 axis plays a key role in regulating autophagic cell death in cardiovascular tissues; moreover, it may be a potential target for novel therapeutic strategies for treating myocardial infarctions [52].

#### 3.2.9. LncRNA Galont

LncRNA Galont expression was significantly upregulated in A/R-treated cardiomyocytes. Overexpression of Galont can aggravate cardiomyocyte autophagy and increase cell death in response to A/R stimuli, whereas Galont knockdown had the opposite effect. Galont was confirmed to directly bind with miR-338, a novel suppressor of autophagy. *ATG5* is a gene targeted by miR-338, and Galont is positively correlated to *ATG5* expression. Thus, the existing evidence suggests that the Galont/miR-338/ATG5 axis plays an important role in promoting A/R-induced autophagy in cardiomyocytes [53].

#### 3.2.10. LncRNA 2810403D21Rik/Mirf

LncRNA 2810403D21Rik/Mirf expression was significantly upregulated in cardiomyocytes treated with H_2_O_2_ and in the heart tissue from the mouse model of MI. LncRNA 2810403D21Rik/Mirf acts as a ceRNA that competitively binds to and affects the activity of miR-26a. miR-26a also modifies the activity of the downstream target Usp15, which can inhibit mitochondrial autophagy by activating the PINK1/PRKN pathway. Silencing 2810403D21Rik/Mirf and overexpressing miR-26a enhanced cell viability and mitigated myocardial injury by increasing the expression of autophagy-related proteins. Further, overexpression of 2810403D21Rik/Mirf suppresses autophagy and exacerbates myocardial injury by limiting the signaling capacity of the miR-26a/Usp15 axis. Therefore, modulating the impact of the 2810403D21Rik/Mirf–miR-26a/Usp15 axis on autophagy can promote a cardioprotective effect in response to a MIRI [54].

#### 3.2.11. LncRNA CAIF

LncRNA CAIF expression was significantly downregulated in H_2_O_2_-treated cardiomyocytes as well as the mouse model of I/R. CAIF can directly bind to the p53 protein and block p53-mediated transcription of *MYOCD*, which encodes myocardin. Myocardin contributes to the induction of the autophagic process and death of cardiomyocytes in the heart during the I/R-induced injury. Knockdown of CAIF can aggravate H_2_O_2_-induced cardiomyocyte autophagy, whereas myocardin overexpression augments the size affected from a MIRI. Moreover, CAIF inhibits autophagy and attenuates myocardial injury in the heart by targeting the p53/myocardin-dependent autophagy pathway [55].

### 3.3. LncRNAs and Necrosis

Cardiomyocyte necrosis is a major pathological event in an AMI. Defining features of necrosis include an increase in cell size from cytoplasmic and mitochondrial swelling, karyolysis, loss of ribosomes from the endoplasmic reticulum, and plasma membrane disruption, all of which initiate inflammation [24, 56]. Mitochondria play an important role in the execution of programmed necrotic cell death, especially in cardiomyocytes, through two mechanisms: mitochondrial permeability transition- (MPT-) dependent necrosis and death receptor-dependent necrosis (necroptosis). Necrosis usually destroys the inner mitochondrial membrane (IMM), thereby inducing the opening of mitochondrial permeability transition pores (MPTPs), which depletes ATP and the plasma membrane loses its integrity [57, 58]. Necroptosis can be induced by triggering several molecules, primarily through death receptors—e.g., TNFR, FasR, and TNF-related apoptosis-inducing ligand receptor (TRAIL-R)—that are stimulated by cytokines from the TNF family and Toll-like receptors (TLRs) [24, 56].

#### 3.3.1. LncRNA NRF

LncRNA NRF expression was significantly upregulated in H_2_O_2_-treated H9c2 cells and the mouse model of I/R. LncRNA NRF directly binds to miR-873, which targets RIPK1 and RIPK3, thereby inhibiting necrosis in cardiomyocytes. Knocking down NRF inhibits necrosis in cardiomyocytes and reduces the infarct size in the I/R mouse model. p53 regulates NRF expression at the transcriptional level and is involved in cardiomyocyte necrosis via miR-873. These results demonstrate that p53 targets NRF–miR-873 and the RIPK1/RIPK3 axis, which play key roles in regulating cardiomyocyte necrosis during a MIRI [59].

#### 3.3.2. LncRNA H19

LncRNA H19 expression decreased in H_2_O_2_-treated H9c2 cells. LncRNA H19 can directly bind to miR-103/107 to promote the expression of Fas-associated protein with death domain (FADD). FADD binds to RIPK1, thereby inhibiting cardiomyocyte necrosis by disrupting the formation of the RIPK1/RIPK3 complex. Inducing H19 expression can inhibit cardiomyocyte necrosis; moreover, H_2_O_2_ induces FADD downregulation. Collectively, these results reveal possible links among H19, miR-103/107, FADD, and RIPK1/RIPK3, which may collectively regulate cardiomyocyte necrosis during a MIRI [60].

### 3.4. LncRNAs and Oxidative Stress

At the onset of reperfusion, the level of myocardial tissue oxygenation increases after restoring blood flow. However, when in a reduced state, respiratory chain complexes damage the mitochondrial electron transport chain, which generates a high level of ROS and leads to ischemia [61, 62]. ROS includes H_2_O_2_, hydroxyl radicals, and superoxide anions. The primary enzyme systems involved in ROS production during a MIRI are xanthine oxidase, NADPH oxidase, the mitochondrial electron transport chain, and uncoupled nitric oxide synthase [63]. Oxidative stress occurs when the antioxidant system is overwhelmed by ROS overproduction. ROS-mediated mitochondrial dysfunction and the sequence of biochemical events following reperfusion of the ischemic area are the key pathological features of a MIRI. ROS overproduction promotes the oxidation of DNA and proteins as well as and lipid peroxidation, thereby causing alterations in membrane permeability that structurally and functionally damage cells [62, 64, 65].

#### 3.4.1. LncRNA Gpr19

The expression of the LncRNA Gpr19 is upregulated in the mouse model of AMI and in neonatal rat ventricular cardiomyocytes (NRCMs) exposed to OGD/R. Gpr19 targets miR-324-5p, a miRNA that negatively regulates Mtfr1 expression at the transcriptional and translational levels. The function of Mtfr1 is primarily related to the IMM. Overexpression of Mtfr1 aggravates oxidative stress in NRCMs induced via OGD/R. Further, suppression of Gpr19 inhibits oxidative stress and attenuates myocardial injury through the miR-324-5p/Mtfr1 axis [66].

#### 3.4.2. LncRNA ROR

LncRNA ROR is highly expressed in I/R-treated H9c2 and HCM cells. Overexpression of ROR can aggravate oxidative stress in response to a MIRI by promoting NADPH oxidase activity, ROS production, and NOX2 protein levels. p38/MAPK inhibitors can rescue LncRNA-ROR-induced cell viability and reduce cell apoptosis in both H9c2 and HCM cells. Further, LncRNA ROR can activate oxidative stress pathways and induce myocardial apoptosis by regulating p38/MAPK signaling [67].

#### 3.4.3. LncRNA HOTAIR

LncRNA HOTAIR expression was significantly downregulated in H_2_O_2_-treated H9c2 cells. HOTAIR modulates the expression of matrix metalloproteinases-2 (MMP2) by binding miR-125 in H9c2 cells when subjected to oxidative stress. Suppression of HOTAIR aggravates cardiomyocyte apoptosis induced by oxidative stress via the HOTAIR/miR-125/MMP2 axis [68].

#### 3.4.4. LncRNA FTX

LncRNA FTX expression is significantly downregulated in the mouse model of I/R and in H_2_O_2_-treated cardiomyocytes. FTX sponges miR-29b-1-5p, which in turn regulates its activity. miR-29b-1-5p promotes H_2_O_2_-induced cardiomyocyte apoptosis by inhibiting the downstream target *Bcl2l2*. Overexpression of FTX inhibits H_2_O_2_-induced cardiomyocyte apoptosis by regulating the miR-29b-1-5p/*Bcl2l2* axis [69].

### 3.5. LncRNAs and Inflammation

Inflammation is an important contributor to the pathophysiology of a MIRI. The I/R-induced injury can activate the inflammatory response by promoting the release of cytokines, chemokines, and other proinflammatory factors [70]. The extent of the inflammatory response to a MIRI can determine the infarct size and subsequent LV remodeling [71, 72]. During the postischemic phase, intracellular content released by necrotic cardiomyocytes initiates an intense inflammatory response by activating innate immune responses. During the early reperfusion phase, cardiomyocytes increase the expression of cytokines and adhesion molecules, which prompt the recruitment of neutrophils and other leukocytes to the infarcted myocardium [73]. As the adherent leukocytes transmigrate into these infarcted zones, they disrupt the microvascular barrier and cause hyperpermeability, leading to myocardium edema that increases the diffusion distance for oxygen and nutrients. Neutrophils are the most abundant among infiltrating inflammatory cells during the reperfusion stage. Notably, they impair endothelial function, activate platelets leading to coronary microembolization, and cause the myocardium no-reflow phenomenon. The infiltration of neutrophils induces a reperfusion injury that amplifies the cellular damage initiated by ischemia [71, 74].

#### 3.5.1. LncRNA KCNQ1OT1

The expression of KCNQ1OT1 was significantly upregulated in OGD/R-induced H9C2 cells. Suppression of KCNQ1OT1 reduces the expression of inflammatory factors (e.g., TNF-*α*, IL-6, and IL-1b) by regulating adiponectin receptors, namely, *AdipoR1*, in H9C2 cells subjected to OGD/R treatment. Moreover, KCNQ1OT1 suppression also inhibits activating the p38/MAPK/NF-*κ*B signaling pathway. Suppression of KCNQ1OT1 may exert protective effects against a MIRI by regulating *AdipoR1*, which in turn affects p38/MAPK/NF-*κ*B signaling [75].

#### 3.5.2. LncRNA NEAT1

LncRNA NEAT1 expression is upregulated in H_2_O_2_-treated H9C2 cells and in the mouse model of I/R. NEAT1 was identified as a functional sponge of miR-495-3p that negatively regulates its expression. miR-495-3p blocks the activation of the inflammation process induced via H_2_O_2_ treatment by downregulating its target, *MAPK6*. Thus, the NEAT1/miR-495-3p/*MAPK6* axis plays a crucial role in activating inflammation and aggravating cardiomyocyte injury during a MIRI [76].

#### 3.5.3. LncRNA H19

LncRNA H19—a precursor of miR-675 that regulates multifarious target genes posttranslationally and is involved in different biological processes—was upregulated in cardiomyocytes exposed to OGD/R. Silencing H19 reduces the production of proinflammatory cytokines and inhibits apoptosis in cardiomyocytes following exposure to OGD/R by downregulating the levels of p-I*κ*B-*α* and p-p65. However, the effect of H19 inhibition was partially reversed when miR-675 was overexpressed. Moreover, miR-675 could directly suppress the expression of PPAR*α*, as demonstrated with dual-luciferase reporter assays and software-based analysis. PPAR*α* exerts an anti-inflammatory effect by suppressing the NF-*κ*B signaling pathway. Therefore, H19 inhibition can markedly improve cardiac structure and function in response to a MIRI via the H19/miR-675/PPAR*α* signaling axis [77].

#### 3.5.4. LncRNA Mirt1

Mirt1, a LncRNA mainly expressed in cardiac fibroblasts, was upregulated in both the myocardium of AMI-induced mice and cardiac fibroblasts that were subjected to hypoxia. High Mirt1 levels contribute towards LV dysfunction in AMI-induced mice, whereas its knockdown could decrease the myocardial infarct size, improve cardiac functions, and potentially limit the inflammatory response. Knockdown of Mirt1 inhibits cardiomyocyte apoptosis and reduces the production of inflammatory cytokines by suppressing NF-*κ*B signaling under hypoxic conditions *in vitro*. These data suggest that Mirt1 knockdown plays a protective role in response to an AMI by inhibiting NF-*κ*B activation [78]. Another study reported that Mirt1 expression levels significantly increased in myocardial tissue of aged diabetic I/R rats. This study also found that the protective effects of Mirt1 inhibition in these rats may result from the inhibition of NF-*κ*B activation by attenuating the inflammatory response, which would normally induce apoptosis and further aggravate the damage to cardiac tissues [79].

#### 3.5.5. LncRNA Gm2691

LncRNA Gm2691 expression was markedly downregulated in the rat model of I/R and in hypoxia-treated NRCMs. Overexpression of Gm2691 significantly decreases IL-6, TNF-*α*, and IL-8 production in hypoxia-induced NRCMs. Hypoxia treatment in NRCMs can inhibit p-AKT expression, whereas the Gm2691 reverses this effect. Thus, LncRNA Gm2691 exerts antiapoptotic and anti-inflammatory effects by regulating the PI3K/AKT signaling pathway [80].

### 3.6. LncRNAs and Mitochondrial Dysfunction

Mitochondrial dysfunction is considered as the prominent pathological feature of a MIRI; therefore, it has become a key regulator of its inhibition. Mitochondria synthesize approximately 90% of ATP in cardiomyocytes [81, 82]. Mitochondrial dysfunction can cause the imbalance of calcium homeostasis, excess production of ROS, impaired cellular energy metabolism and ATP production, and opening of MPTPs, which can culminate in cell death [83]. Numerous studies have demonstrated that an imbalance between mitochondrial fission and fusion contributes towards mitochondrial dysfunction in response to a MIRI. During the early ischemic period, mitochondrial fusion leads to the generation of a disproportionately large amount of ATP, while sustained ischemia induces excessive mitochondrial fission. During the reperfusion period, excessive ROS and calcium overload exacerbate the activation of mitochondrial fission. Mitochondrial fission generates smaller mitochondrial fragments; however, mitophagy insufficiently eliminates damaged mitochondria. Thus, mitochondrial fission results in a higher susceptibility of opening MPTPs, activating apoptotic pathways by releasing cytC, and activating caspases, all of which result in cell death following myocardial reperfusion [84–86].

#### 3.6.1. LncRNA CARL

LncRNA CARL is expressed in both the nucleus and the cytoplasm. The expression of CARL was downregulated in cardiomyocytes following anoxia treatment. CARL acts as an endogenous sponge of miR-539, thereby negatively regulating its levels and activity. miR-539 targets and regulates PHB2 expression, which plays an important role in regulating mitochondrial morphology. Overexpression of CARL attenuates mitochondrial fission and cell death during a MIRI by sponging miR-539 as well as upregulating PHB2 expression *in vitro* and *in vivo*. Combined, these data suggest that the CARL/miR-539/PHB2 signaling is crucial in affecting mitochondrial dynamics and cell apoptosis during a MIRI [87].

#### 3.6.2. LncRNA MDRL

LncRNA MDRL is 1,039 nucleotides and is expressed in both the nucleus and the cytoplasm. MDRL expression was downregulated in A/R-treated cardiomyocytes. The bioinformatics program RNAhybrid revealed that MDRL carries a target site for miR-361. miR-361 promotes mitochondrial fission and apoptosis following A/R treatment by inhibiting the processing of pri-miR-484 by Drosha into pre-miR-484. MDRL acts as an endogenous sponge for miR-361 to regulate mature miR-484 levels. In an animal model, MDRL knockdown attenuated mitochondrial fission, cardiomyocyte death, and myocardial infarction size in response to I/R. These findings suggest that MDRL targets miR-361/miR-484 in the mitochondrial fission cascade and apoptosis during a MIRI, thereby revealing potential targets for treating cardiac disease [88].

### 3.7. LncRNAs and Calcium Overload

Calcium homeostasis is disturbed during myocardial ischemia-reperfusion. Excessive accumulation and leakage of intracellular calcium lead to calcium overload, which exacerbates a MIRI [89, 90]. Cytosolic and mitochondrial calcium overload begins at ischemia and is aggravated during reperfusion. At the onset of ischemia, the Na^+^/H^+^ exchanger (NHE) is activated and transports H^+^ out of the cytosol in exchange for Na^+^. During reperfusion, NHE activity is accelerated, leading to additional accumulation of intracellular Na^+^. As a result, a high Na^+^ concentration activates the Na^+^/calcium exchanger (NCX), thereby leading to intracellular calcium overload [91]. Alternatively, more calcium influx through L-type calcium channels and the sarcoplasmic reticulum uptake of calcium from the cytoplasm is inhibited, which further exacerbates the calcium overload in response to the I/R [92]. During reperfusion, disruption of the mitochondrial membrane potential accelerates energy depletion, thereby resulting in mitochondrial calcium overload and ROS overproduction. In addition, disrupting the mitochondrial membrane potential also results in ATP synthase behaving as an ATPase, thereby accelerating energy depletion in response to the ischemic insult. Large amounts of calcium accumulated in cells inhibit ATP synthesis in mitochondria, leading to the dysfunctional processing of energy metabolism. These processes can trigger MPTPs to open, leading to the breakdown of respiratory chain coupling, mitochondrial membrane potential disorder, and eventually the induction of either necrotic- or apoptotic-mediated cell death [93].

#### 3.7.1. LncRNA ZFAS1

LncRNA ZFAS1, an antisense LncRNA to the 5′ end of the protein-coding gene *Znfx1*, has been identified as an independent predictor of an AMI [94]. Calcium homeostasis is closely related to cardiac contractility, and abnormal intracellular calcium handling might contribute to the impairment of cardiac contractile function caused by ZFAS1 in a MIRI. Sarcoplasmic reticulum calcium-ATPase 2a (SERCA2a) is a key protein involved in the reuptake of calcium into the sarcoplasmic reticulum during diastole, thereby regulating calcium homeostasis in cardiomyocytes. ZFAS1 expression is markedly elevated in the myocardium of mice with an AMI. Knockdown of endogenous ZFAS1 improved cardiac function as the ejection fraction (EF), and fractional shortening (FS) resumed to almost normal levels in the mouse model of myocardial infarction. ZFAS1 overexpression in normal mice could impair cardiac function as EF and FS both decreased, thereby promoting an enlarged LV internal dimension at end-diastole (LVIDd) and LV internal dimension at systole (LVIDs), which is similar to the pathological process that occurs in response to a MIRI. Additionally, ZFAS1 conferred a negative effect on SERCA2a by repressing its expression at the transcriptional level. ZFAS1 deleteriously impacted the dynamic influx of calcium, thereby leading to intracellular calcium overload in cardiomyocytes, which might be the underlying mechanism of its proapoptotic property. Moreover, it was found that NFATc2 served as a transactivator to promote ZFAS1 expression [95]. Another study found that ZFAS1 activated the mitochondrial apoptosis pathway in the mouse model of myocardial infarction. Additionally, ZFAS1 knockdown relieved mitochondrial swelling and decreased mitochondrial membrane potential in hypoxia-treated cardiomyocytes. ZFAS1 induced cardiomyocyte apoptosis by inhibiting SERCA2a, thereby causing intracellular calcium overload. Therefore, knocking down ZFAS1 protects cardiac tissues against MIRI-induced dysfunction and cardiomyocytes against hypoxia treatment-induced apoptosis [96].

## 4. Clinical Utility of LncRNAs

### 4.1. Biomarkers

Due to the high mortality rate of patients with an AMI, early detection and treatment can considerably improve the prognosis of these patients. Finding a myocardial biomarker with high specificity and sensitivity has always been a hotspot in the cardiovascular research field. LncRNAs have a high degree of tissue specificity and are relatively stable secondary structures present within samples of plasma, serum, urine, and other body fluids. Thus, LncRNAs can be novel biomarkers to diagnose and predict the prognosis of several diseases [97, 98] (Table 2).

An increasing number of clinical studies have measured changes in circulating LncRNAs of AMI patients and have investigated their roles as novel diagnostic biomarkers for AMIs. Lu et al. discovered seven LncRNAs by analyzing LncRNA expression in genomes from 52 acute coronary syndrome patients; moreover, they compared the LncRNA expression pattern between patients with a myocardial infarction or an unstable angina. The overall classification exhibited 90.38% accuracy, 100% sensitivity, and 68.75% specificity. These results suggest that these novel, functional LncRNAs may be candidate diagnostic biomarkers and potential therapeutic targets [99]. Li et al. reported LncRNA expression patterns using two microarray datasets from patients with AMI and healthy samples from the Gene Expression Omnibus (GEO) database. They found that 11 differentially expressed LncRNAs had a diagnostic value in AMI patients and may be used as potential biomarkers for early diagnosis of AMIs [100].

Gao et al. found that HOTAIR expression levels in the serum of AMI patients were significantly decreased compared with that of healthy controls. Thus, the LncRNA HOTAIR may serve as a sensitive predictor for AMI diagnosis [101]. Li et al. found that the LncRNA LIPCAR may serve as a potential biomarker for ST-segment elevation myocardial infarction. The extent of LncRNA elevation also could reflect the severity of a coronary stenosis. Multivariate Cox regression analysis revealed that the Gensini score and LIPCAR levels were independent predictors of major adverse cardiac events following an AMI [102]. Yan et al. found that LncRNA UCA1 levels decreased in plasma samples from patients with early stage AMI and increased in 72–96 h following AMI onset. They proposed that the circulating concentration of LncRNA UCA1 may serve as a potential biomarker for diagnosing AMI [103]. Vausort et al. reported that LncRNAs ANRIL and KCNQ1OT1 within the peripheral blood of patients with AMI can be used as predictors of LV dysfunction following a MIRI [104]. Wang et al. found that LncRNA CHAST expression was upregulated in patients with AMI at 24 h but substantially decreased 3 d following AMI onset, indicating that CHAST may serve as a potential biomarker for diagnosing AMI. Moreover, circulating CHAST levels within 24 h can be used to predict cardiac contractile function in patients with AMI independently [105]. Wang et al. found that the LncRNAs H19, MIAT, and MALAT1 indicated an adverse outcome from an AMI. Peripheral blood mononuclear cell- (PBMC-) derived levels of the LncRNAs H19, MIAT, and MALAT1 were increased in AMI patients suggesting that they may be a useful diagnostic biomarker for AMI [106]. Zhang et al. found that LncRNA ZFAS1 expression was downregulated, but LncRNA CDR1AS expression was upregulated in the peripheral blood of patients with AMI. They also found that either ZFAS1 or CDR1AS correlated with an AMI; however, the combination of the two or their reciprocal changes elicited greater sensitivity and specificity to accurately predict, thereby indicating that they are superior biomarkers for AMI [94].

In addition, there are several studies demonstrating that LncRNAs also serve as novel diagnostic biomarkers of coronary artery disease (CAD). Zhang et al. found that increased plasma levels of H19 and LIPCAR were associated with an increased risk of CAD and may be considered as novel biomarkers for diagnosing CAD [107]. Interestingly, the polymorphisms of H19 are associated with the risk and severity of CAD in a Chinese population, which might provide new markers for the prevention and early intervention of CAD [108]. Li et al. identified aberrantly expressed LncRNAs in CAD patients by analyzing LncRNA expression in the transcriptomes of 93 CAD patients and 48 healthy controls. The authors identified ENST00000444488.1 and uc010yfd.1 as novel LncRNA biomarkers for diagnosing CAD [109]. A recent study found that the LncRNA OTTHUMT00000387022 in PBMCs can be recognized as a novel biomarker due to its high sensitivity and specificity for diagnosing CAD [110].

Although the prospect of using LncRNAs as new diagnostic and prognostic biomarkers in cardiovascular disease is advancing, there are some limitations. For example, some LncRNAs need to be revalidated in a multicenter clinical study with a large sample size to ensure their diagnostic potential as a biomarker. Additionally, some previous clinical studies and samples related lack follow-up information; therefore, the predictive value of the LncRNA cannot be fully assessed. Recently, an in-depth study on the epigenetic mechanisms of differentially expressed LncRNAs in response to a MIRI is in progress; however, the standardized protocol of the test operation has not been confirmed. For instance, from acquiring biological fluids to recovering a small amount of nucleic acids, the quantification of these samples also presents a challenging technical query that should be further explored.

### 4.2. Therapy

With the recent advancing research on MIRI mechanisms, new therapeutic strategies have been developed to help limit the myocardium injury following an AMI. To reduce the infarct size, preserve the LV function, and improve clinical outcomes of AMI patients, LncRNAs have emerged as important regulatory molecules in the pathological progression of MIRIs [111]. Recent studies have reported that upregulating or downregulating the expression level of LncRNA can relieve a MIRI in mice. These trials also indicated multiple LncRNAs are likely to emerge as novel therapeutic candidates to target and treat AMI (Table 2).

In the I/R mouse model, the intracoronary delivery of adenovirus-expressed LncRNAs MDRL [88] or CARL [87] notably inhibited mitochondrial fission, myocardial cell death, and myocardial infarction sizes and preserved cardiac function. Moreover, silencing any of the following LncRNAs, namely, APF [52], Meg3 [112], NEAT1 [33], H19 [77], MALAT1 [29], NRF [59], Mirt1 [78], or ZFAS1 [95], can reduce myocardial infarction size and preserve cardiac function.

In the rat I/R model, HOTAIR overexpression could suppress inflammation and myocardial cell apoptosis, as well as reduce myocardial infarction sizes [113]. Additionally, inhibiting either Mirt1 [79] or ZFAS1 [114] suppressed myocardial cell apoptosis, reduced myocardial infarction sizes, and improved cardiac function following an I/R-induced injury.

Although the results of using LncRNA therapeutics in animal experimental studies are promising, several limitations persist. Interestingly, owing to their therapeutic potential in preventing and treating a MIRI, gene therapy has become a promising approach to effectively target LncRNAs. However, our present understanding is limited to the animal models, and the safety and effectiveness of its clinical application require further research.

## 5. Conclusions and Perspectives

We have begun the era of “noncoding.” We look forward to seeing more reports of noncoding RNAs, particularly LncRNAs, that regulate various basic biological processes including cardiovascular biology and related diseases. LncRNAs have the potential to be biomarkers for clinical diagnoses and targets for gene therapy. However, LncRNA-based research in MIRIs is still in its infancy, and further investigation is required to fully elucidate the role of LncRNAs in MIRIs. For example, the majority of available evidence linking LncRNAs and MIRIs is limited *in vitro* data; moreover, nonconservative sequences and limitations of some experimental methods hinder the progress of *in vivo* experiments. Whether modifying LncRNAs could fully regulate the cellular response to a MIRI, there are relatively few studies on LncRNAs in this field. These hurdles must be overcome before LncRNAs can be used in clinical trials. With the innovation and advancement of LncRNA-based research, their mechanistic role in MIRIs is expected to be elucidated. Therefore, LncRNAs would be ideal and novel targets for the diagnosis and treatment of patients with AMI.

## Figures and Tables

**Table 1:** The role of LncRNAs in MIRI.

Mechanisms	LncRNA	Mechanism	Species	Expression	Functions	Ref.
Apoptosis	MALAT1	*β*-Catenin protein	Rat	Up	Knockdown of MALAT1 inhibits the cardiomyocyte apoptosis and improves cardiac function	[26]
		miR-122/AKT/GSK-3*β*/*β*-catenin	RPCM/H9c2	Up	Knockdown of MALAT1 inhibits the cardiomyocyte apoptosis	[27]
		miR-200a-3p/PDCD4	AC16/mice	Up	Promote cardiomyocyte apoptosis	[28]
		miR-320/PTEN	NMCM/mice	Up	Promote cardiomyocyte apoptosis and aggravate myocardial injury	[29]
	PFL	HSP-20/Bax/Bcl-2	Rat	Up	Knockdown of PFL inhibits apoptosis and reduces the MI area	[30]
	HIF1A-AS1	miR-204/SOCS2	Mice	Up	Silence of HIF1A-AS1 inhibits cardiomyocyte apoptosis and alleviates ventricular remodeling	[31]
	NEAT1	miR-520a	H9c2/rat	Up	Knockdown of NEAT1 inhibits cardiomyocyte apoptosis	[32]
		MAPK	H9c2/mice	Up	Promote cardiomyocyte apoptosis	[33]
	Meg3	miR-7-5p/PARP1	H9c2/rat	Up	Promote cardiomyocyte apoptosis and myocardial damage	[34]
	MIAT	NF-*κ*B/PUMA	H9c2/mice	Up	Silence of MIAT inhibits cardiomyocyte apoptosis	[35]
	XIST	miR-130a-3p/PDE4D	AC16	Up	Knockdown of XIST inhibits apoptosis and promotes cell survival	[36]
	AK123484	PARP and caspase-3	H9c2/rat	Up	Knockdown of AK123483 inhibits apoptosis	[37]
	BDNF-AS	BDNF/VEGF/Akt	NMCM	Up	Promote cardiomyocyte apoptosis	[38]
	H19	miR-877-3p/Bcl-2	NMVC/mice	Down	H19 alleviates cardiomyocyte apoptosis	[39]
	UCA1	p27	RPCM/rat	Down	UCA1 inhibits cardiomyocyte apoptosis	[40]
	FAF	PI3K/AKT/FGF9/FGFR2	NRCM/rat	Down	FAF inhibits cardiomyocyte apoptosis	[41]
Autophagy	TUG1	miR-142-3p/HMGB1 and Rac1	NMCM/mice	Up	Knockdown of TUG1 inhibits excessive autophagy	[46]
	XIST	miR-133a/SOCS2	H9c2/mice	Up	Knockdown of XIST inhibits cardiomyocyte autophagy	[47]
	HRIM	LC3	H9c2/rat	Up	Inhibition of HRIM reduces excessive autophagy	[19]
	AK139328	miR-204-3p	Mice	Up	Silence of AK139328 inhibits cardiomyocyte autophagy	[48]
	AK088388	miR-30a/Beclin-1/LC3-II	HL-1	Up	Enhanced autophagy and promote cardiomyocyte injury	[49]
	FOXD3-AS	NF-*κ*B/iNOS/COX2	H9c2	Up	Aggravate cardiomyocyte injury via promoting autophagy	[50]
	RMRP	miR-206/ATG3	H9c2/rat	Up	Promote cardiomyocyte autophagy	[51]
	APF	miR-188-3p/ATG7	NMCM/mice	Up	Increase autophagy and myocardial infarction	[52]
	Galont	miR-338/ATG5	NMCM	Up	Promote autophagy and cell death	[53]
	2810403D21Rik/Mirf	miR-26a/Usp15	NMCM/mice	Up	Suppress autophagy and exacerbate myocardial injury	[54]
	CAIF	p53/Myocardin	NMCM/mice	Down	Suppress autophagy and attenuate myocardial infarction	[55]
Necrosis	NRF	miR-873/RIPK1/RIPK3	H9c2/mice	Up	Knockdown of NRF inhibits necrosis and reduces infarction size	[59]
	H19	miR-103/107/FADD	H9c2	Down	Alleviate cardiomyocyte necrosis	[60]
Oxidative stress	Gpr19	miR-324-5p/Mtfr1	Mice/NRCM	Up	Suppression of Gpr19 inhibits oxidative stress	[66]
	ROR	p38/MAPK	H9c2/HCM	Up	Aggravate oxidative stress	[67]
	HOTAIR	miR-125/MMP2	H9c2	Down	Suppression of HOTAIR aggravates oxidative stress-induced cardiomyocyte injury	[68]
	FTX	miR-29b-1-5p/Bcl2l2	NMCM/mice	Down	Overexpression of FTX inhibits oxidative stress	[69]
Inflammation	KCNQ1OT1	p38 MAPK/NF-*κ*B	H9c2	Up	Suppression of KCNQ1OT1 reduces inflammation	[75]
	NEAT1	miR-495-3p/MAPK6	H9C2/mice	Up	Activating inflammation	[76]
	H19	miR675/PPAR*α*	NMCM/mice	Up	Knockdown of H19 inhibits inflammation, improves cardiac structure and function	[77]
	Mirt1	NF-*κ*B	NMCM/mice	Up	Knockdown of Mirt1 inhibits inflammation and reduces infarction size	[78]
		NF-*κ*B	Rat	Up	Inhibition of Mirt1 relieves inflammatory injury	[79]
	Gm2691	PI3K/Ak	NRCM/rat	Down	Anti-inflammation	[80]
Mitochondrial dysfunction	CARL	miR-539/PHB2	NMCM/mice	Down	Attenuate mitochondrial fission and cell death	[87]
	MDRL	miR-361/miR-484	NMCM/mice	Down	Attenuate mitochondrial fission and apoptosis	[88]
Calcium overload	ZFAS1	SERCA2a	NMCM/mice	Up	Aggravate calcium overload	[94–96]

**Table 2:** Clinical utility of LncRNA.

Clinical utility	Model	Expression	LncRNA	Ref.
Biomarkers	AMI	Up	RP11-68I3.11, AC068831.6	[99]
		Up	LOC145474, LOC100129518, BRE-AS1, MIR22HG, MIR3945HG, ATP2B1-AS1, CATIPAS1, LINC00528	[100]
		Up	LIPCAR	[102]
		Up	UCA1 (72–96 h after onset of AMI)	[103]
		Up	KCNQ1OT1	[104]
		Up	CHAST	[105]
		Up	H19, MIAT, MALAT1	[106]
		Up	CDR1AS	[94]
		Down	RP11-133L14.5, PAX8-AS1, RP11-259K15.2, RP11-203M5.8, LINC01254	[99]
		Down	WDR86-AS1, A2M-AS1, and LINC00612	[100]
		Down	HOTAIR	[101]
		Down	UCA1 (early stage of AMI)	[103]
		Down	ANRIL	[104]
		Down	ZFAS1	[94]
	CAD	Up	H19	[107–108]
		Up	LIPCAR	[107]
		Up	ENST00000444488.1	[109]
		Up	OTTHUMT00000387022	[110]
		Down	uc010yfd.1	[109]
Therapy	Mice	Up	MDRL	[88]
		Up	CARL	[87]
		Down	APF	[52]
		Down	Meg3	[112]
		Down	NEAT1	[33]
		Down	H19	[77]
		Down	MALAT1	[29]
		Down	NRF	[59]
		Down	Mirt1	[78]
		Down	ZFAS1	[95]
	Rat	Up	HOTAIR	[113]
		Down	Mirt1	[79]
		Down	ZFAS1	[114]
